# Human insulin modulates α-synuclein aggregation via DAF-2/DAF-16 signalling pathway by antagonising DAF-2 receptor in *C. elegans* model of Parkinson’s disease

**DOI:** 10.18632/oncotarget.27366

**Published:** 2020-02-11

**Authors:** Rizwanul Haque, Shamsuzzama, Lalit Kumar, Tanuj Sharma, Soobiya Fatima, Pooja Jadiya, Mohammad I. Siddiqi, Aamir Nazir

**Affiliations:** ^1^Division of Neuroscience and Ageing Biology, CSIR-Central Drug Research Institute, Lucknow 226 031, India; ^2^Laboratory of Computational Biology and Bioinformatics, Division of Molecular and Structural Biology, CSIR-Central Drug Research Institute, Lucknow 226 031, India; ^3^Academy of Scientific and Innovative Research (AcSIR), Anusandhan Bhawan, New Delhi 110 001, India

**Keywords:** Parkinson’s, human insulin, aggregation, Daf-2, Caenorhabditis elegans (C. elegans)

## Abstract

Insulin-signalling is an important pathway in multiple cellular functions and organismal ageing across the taxa. A strong association of insulin-signalling with Parkinson’s disease (PD) has been proposed but the exact nature of molecular events and genetic associations are yet to be understood. We employed transgenic *C. elegans* strain harboring human α-synuclein::YFP transgene, towards studying the aggregation pattern of α-synuclein, a PD-associated endpoint, under human insulin (Huminsulin®) treatment and DAF-16/DAF-2 knockdown conditions, independently and in combination. The aggregation was increased when DAF-16 was knocked-down independently or alongwith a co-treatment of Human insulin (HumINS) and decreased when DAF-2 was knocked-down independently or alongwith a co-treatment of HumINS; whereas HumINS treatment per se, reduced the aggregation. Our results depicted that HumINS decreases α-synuclein aggregation via DAF-2/DAF-16 pathway by acting as an antagonist for DAF-2 receptor. Knockdown of reported DAF-2 agonist (INS-6) and antagonists (INS-17 and INS-18) also resulted in a similar effect on α-synuclein aggregation. Further by utilizing bioinformatics tools, we compared the differences between the binding sites of probable agonists and antagonists on DAF-2 including HumINS. Our results suggest that HumINS treatment and DAF-16 expression play a protective role against α-synuclein aggregation and its associated effects.

## INTRODUCTION

Neurodegenerative diseases (NDs) are age associated ailments that pose a great challenge for the elderly population. These diseases result from neuronal degeneration leading to altered neurotransmitter signalling and impaired neurobiological functions. NDs include Alzheimer’s disease, Parkinson’s disease (PD) and Amyotrophic-Lateral-Sclerosis, amongst others, each believed to be multifactorial in origin. Over the last four decades there has been great progress with respect to understanding the molecular mechanisms of PD. The inability to interlink the group of abnormalities under a single primary pathogenic mechanism of this disease still exists. However, review of the previous findings elucidates the role of insulin or its related pathways in the production of amyloid-aggregates. Insulin also prevents against neurodegeneration resulting from the initial abnormalities that may finally lead to cognitive damage. Insulin-signalling pathway is one of the most extensively studied and the progression of NDs has been proposed to be linked with this evolutionarily conserved pathway in various model systems [1]. The typical molecular and pathological features of NDs have been associated with the reduced expression of the insulin and IGF genes in human brains [2]. Proteome profiling in postmortem brain tissues from ND patients have shown metabolic perturbations of insulin-signalling in CNS [3, 4]. Furthermore, decreased insulin-signalling is reported to play protective role in neurodegenerative-associated proteotoxicity across species [5, 6]. In addition, insulin-signalling is also widely acknowledged to be acting as a neuro-protectant and assists in growth and neuronal survival [7, 8]. It is perhaps more likely that insulin-signalling or its linked pathways are responsible for the generation of these toxic products. Recently, large number of studies has associated the Type 2 Diabetes Mellitus (T2DM) and its related metabolic outcome with conditions associated with PD [9–14]. Studies provide evidence that alterations in the insulin and insulin-like-growth-factor signalling leads to initial stages of PD [15]. T2DM patients tend to show cognitive impairment which may be attributed to brain insulin deficiency and its resistance [16]. IRS, a downstream signalling molecule in the insulin signalling pathway in mammals, modulates major molecular and biochemical responses through its signalling cascade, thus affecting apoptosis, oxidative conditions, growth, survival, energy metabolism, and cholinergic gene expression [9, 15]. Secretion of insulin-related proteins from neurosecretory cells, governs the regulation of survival and differentiation of neurons and/or functions as neuro- modulators [16]. Besides, it has been already reported that decreased insulin-signalling exerts a protective effect against neurodegenerative-associated proteotoxicity across species [5].

Insulin molecule is conserved and is ubiquitously expressed in vertebrates, insects and nematodes and belongs to a class of secreted proteins of super-family sharing a structural motif stabilized by disulfide linkages [17, 18]. Insulin super-family genes have been identified in invertebrates, including insects, molluscs, and the nematode *Caenorhabditis elegans* (*C. elegans*) [19]. In *C. elegans*, insulin like signalling (ILS) pathway is regulated by ILS ligands namely INS-1 to INS-39. These ligands modulate the activity of a tyrosine kinase receptor, DAF-2; an orthologue of mammalian insulin/IGF receptor [20]. This receptor activates a cascade of protein kinases which ultimately terminates at the phosphorylation of downstream transcription-factor DAF-16 by AKT protein kinase. DAF-2 regulates functions similar to receptor kinases in the insulin-signalling pathway in humans [21]. DAF-2 activation phosphorylates DAF-16 to prevent its nuclear entry and transcription thereof. This inhibits the expression of genes governing metabolism, physiological defense and homeostasis responses [22]. On the contrary, under reduced DAF-2 signalling, DAF-16 is not being able to getphosphorylated and its entry to nucleus is facilitated. *daf-2* mutants show doubled life-span as wild-type animals [23], and the lack of the *daf-16* gene resulted in shortened lifespan [24]. In fact, treatment of insulin directly to *C. elegans* has also been shown to regulate lifespan [25].

This emergence of new concept i.e. modulation of pathological hallmark of NDs by insulin-signalling pathways prompted us to study the relation of insulin and its related pathways in the context of PD. *C. elegans* exhibits many important neuronal components found in humans, including neurotransmitters, ligand receptors, and ion channels [26] and it offers several advantages for studying human CNS diseases. Hence the present study employ *C. elegans* model towards exploring the effect of HumINS on α-syn (α-synuclein) aggregation and further towards dissecting out the pathways involved behind the cause and prevention process.

## RESULTS

### HumINS decreases α-syn aggregation

**Figure 1:**
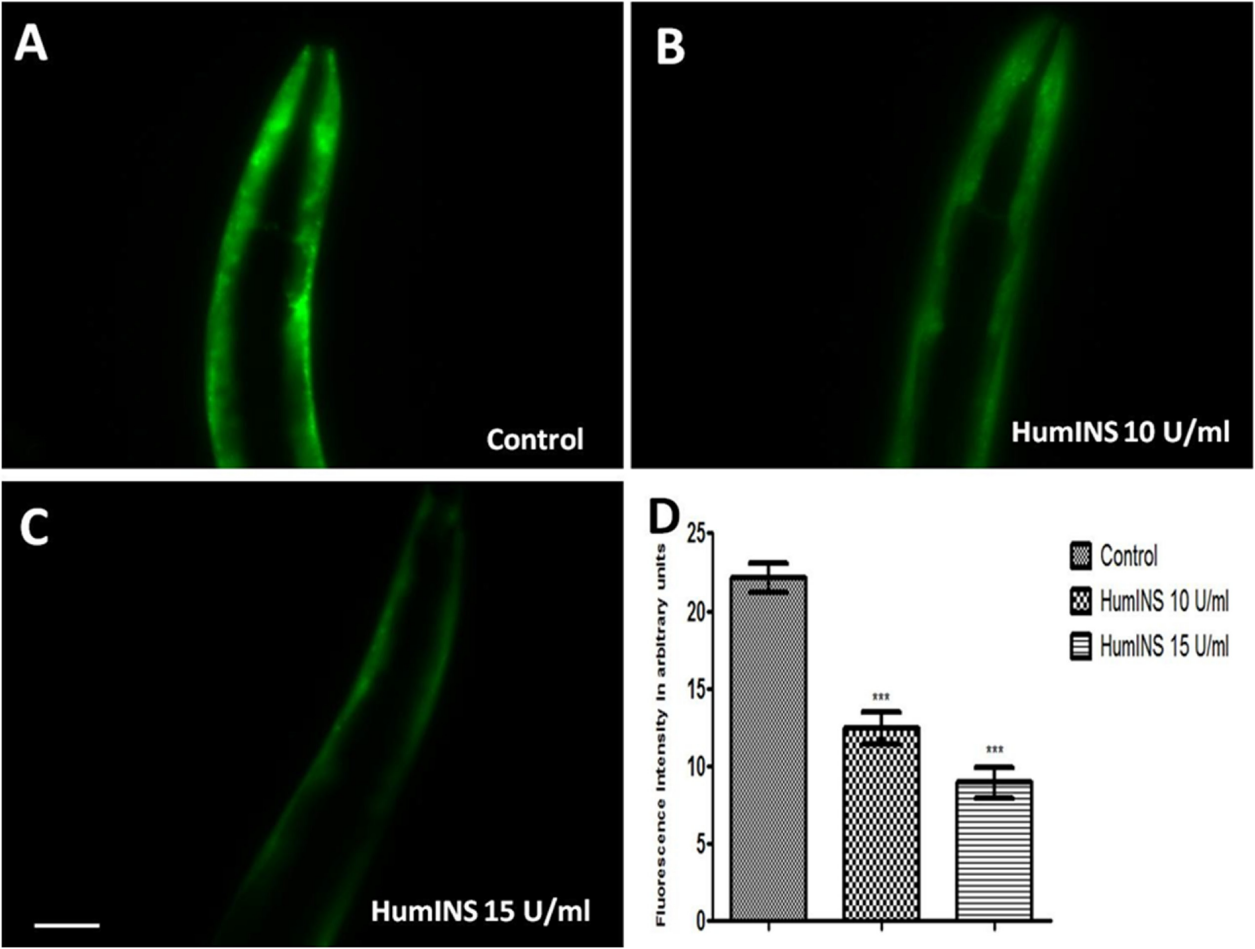
Effect of HumINS on α-syn expression. (**A–C**) The expression of α-syn was assayed in NL5901 transgenic strain of *C. elegans* that expresses human α-syn::YFP transgene in their body wall muscles. The worms were treated with OP50 (control) (A), 10 U/ml HumINS (B), 15 U/ml HumINS (C). (**D**)There was a decrease in the α-syn level. Figure 1D is graphical representation for fluorescence intensity of α-syn::YFP as quantified using Image J software. ^***^ *p* < 0.001, Scale bar, 50 µm.

HumINS is reported to cross bind with the *C. elegans* insulin-receptor DAF-2 which bears high homology with HumINS receptor [20]. Considering the importance of insulin pathway in NDs, we explored the effect of HumINS on α-syn abundance/aggregation. Out of the several hypothesis associated with PD, one is the production of toxic protein that gets deposited and results in toxic assaults. A lot of evidence is accumulating through clinical and epidemiological research suggesting that drugs developed for the treatment of T2DM might be highly beneficial for the treatment of PD and associated diseases [27]. Intranasal administration of exogenous insulin in people with cognitive dysfunction has been proved fruitful, making insulin as an effective therapy against NDs and taking it to the level of pilot clinical studies [28]. In order to examine the effect of HumINS against another parameter of PD i.e. α-syn expression we employed the transgenic *C. elegans* strain, NL5901 [29]. This worm model of PD serves as an ideal model for PD research; it over-expresses human α-syn fused with YFP (yellow fluorescent protein) which easily allows for visual detection of α-syn aggregation by a fluorescent microscope. The representative worm model of PD expresses α-syn in muscles and not in neurons. This may or may not have direct relevance to PD which restrains it from being perfect replica of mammalian PD condition. However, this model has previously been shown to be beneficial in terms of genetic modulations and associated aspects. As described previously, concentration of 10 U/ml and 15 U/ml of insulin was chosen because of its protective effects against neuronal damage and extension of lifespan in the nematode *C. elegans* [25]. The mean fluorescence (GFP) intensity was 22.15 ± 0.91 *N* = 7 arbitrary units in control worms, 12.48 ± 1.04 *N* = 7 arbitrary units in 10 U/ml HumINS treated worms and 8.93 ± 0.97 *N* = 7 in 15 U/ml HumINS treated worms (Figure 1D). 1.77 and 2.48-fold reduction (*p* < 0.001) in α-syn protein expression was observed in 10 and 15 U/ml HumINS exposed worms, respectively. Our finding suggest that the worms from HumINS treatment group exhibited a decrease in the α-syn::YFP fluorescence intensity after treatment with 10 and 15 U/ml HumINS (Figure 1). Further, employing Western blotting we quantified the level of aggregated and soluble form of α-syn. The content of total, aggregated and soluble protein loading onto each well was calculated so as to represent their concentration in total protein fraction extracted from control and 15 U/ml HumINS treated NL5901 worms. We observed that worms of HumINS treated group exhibited lesser aggregation and expression of α-syn as compared to the worms of control group (Figure 5). This clearly demonstrates that HumINS led to decrease in the α-syn aggregation and the protective effect of HumINS was more pronounced in 15 U/ml HumINS treatment.

**Figure 2:**
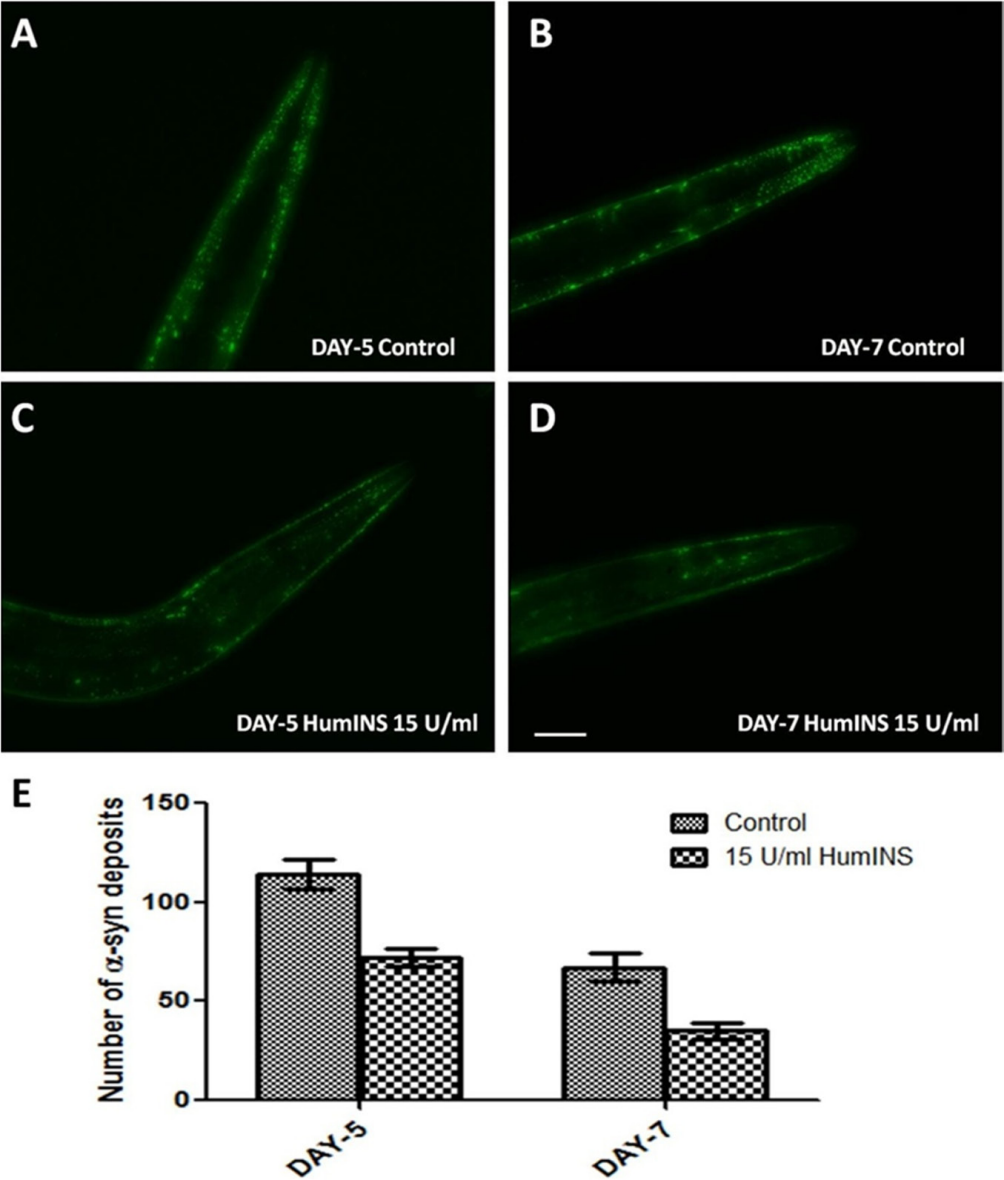
HumINS decreases aging-dependent aggregate accumulation. (**A–D**) The aggregation of α-syn was assayed in NL5901 transgenic strain of *C. elegans* that expresses human α-syn::YFP transgene in their body wall muscles at day 5 and day 7. Representative image (A) represents 5-day-old worms fed on OP50; (B) represents 7-day-old worms fed on OP50; (C) 5-day-old worms treated with 15 U/ml HumINS; (D) 7-day-old worms treated with 15 U/ml HumINS. (**E**) Graphical representation for numbers of α-syn deposits as quantified using DotCount image analysis software. ^***^ *p* < 0.001, Scale bar, 100 µm.

**Figure 3:**
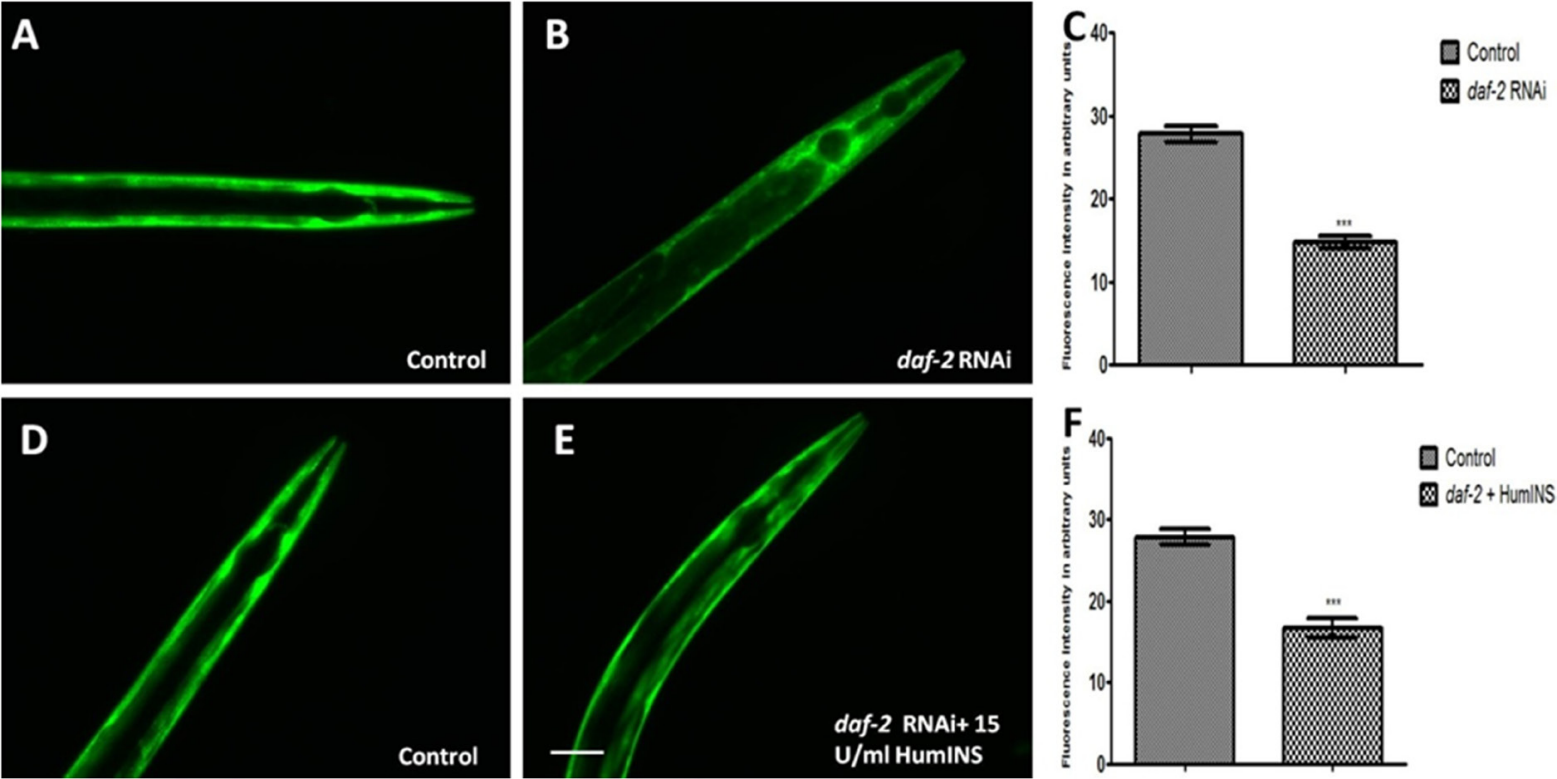
HumINS acts via DAF-2 receptor based signalling in reducing α-syn expression. (**A**, **B**, **D**, **E)** Transgenic *C. elegans* strains expressing human α-syn::YFP were subjected to DAF-2 knockdown and DAF-2 knockdown along with co-treatment of HumINS. The image represents the worms fed on OP50 (control; A), and the worms fed on *daf-2* RNAi bacteria (B). The worms fed on OP-50 (control; D) and worms fed on *daf-2* RNAi bacteria along with 15 U/ml of HumINS (E). The worms of the *daf-2* silenced (B) and double treatment group (E) resulted in decreased α-syn expression as compared to worms of the control group (A, D). Knocking down DAF-2 rendered ILS pathway inactive and the protective effect of HumINS in decreasing α-syn was not synergistic. (**C**, **F**) Graphical representation for fluorescence intensity of α-syn::YFP as quantified using Image J software. ^***^ *p* < 0.001, Scale bar, 50 µm.

**Figure 4:**
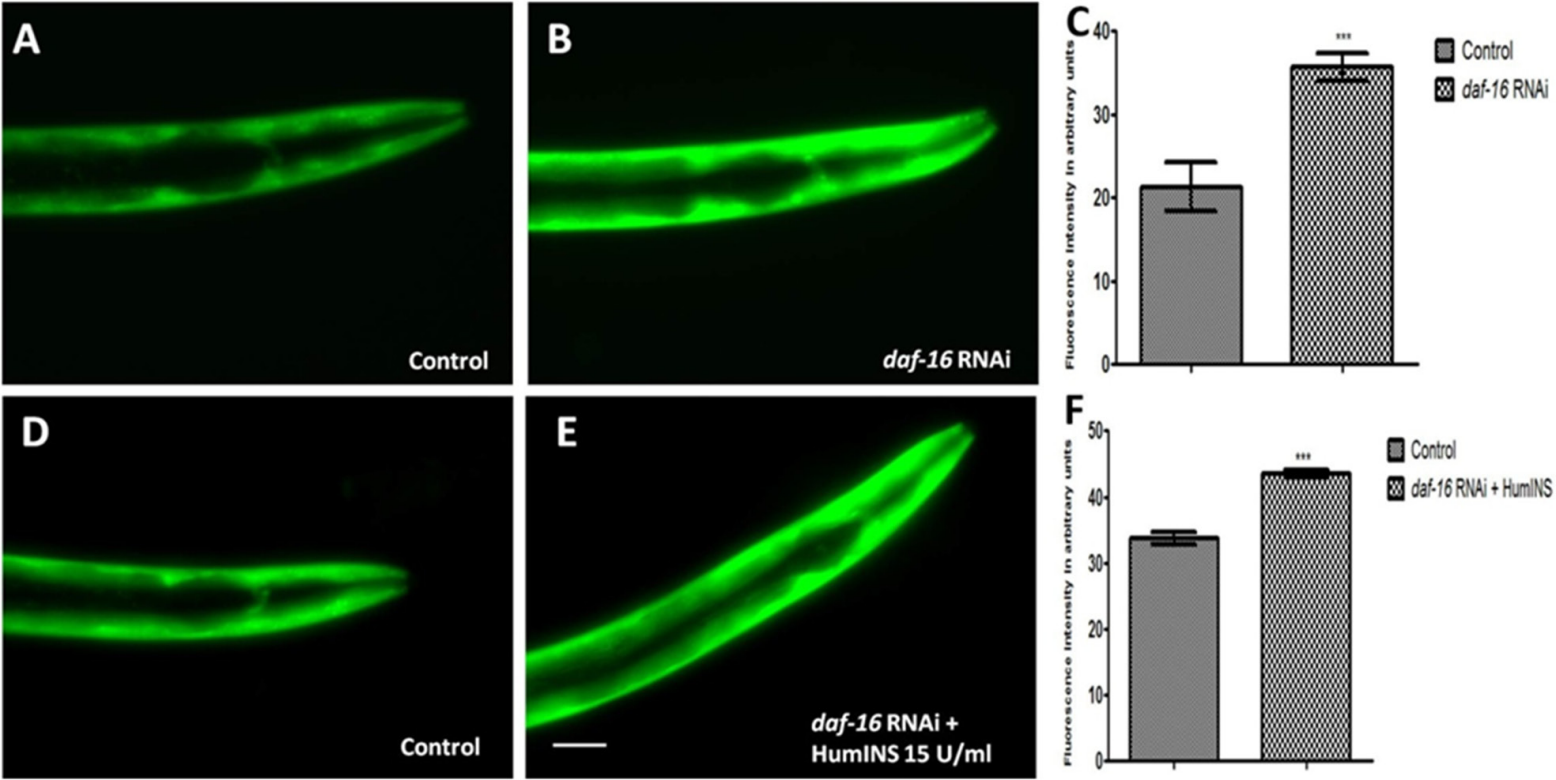
Protective action of HumINS against α-syn expression also involves FOXO transcriptional factor DAF-16 of the *C. elegans* ILS pathway. (**A**, **B**, **D**, **E**) Fluorescent images represent DAF-16 knock-down and double treatment studies of NL5901 transgenic strain of *C. elegans*, in which transgenic worms were subjected to both DAF-16 knockdown and DAF-16 knockdown along with co-treatment of HumINS. The worms were fed on OP-50 (A; control) and bacteria expressing double stranded RNA against *daf-16* gene (B). And worms were fed on OP-50 (D; control) and bacteria expressing double stranded RNA against *daf-16* gene along with co-treatment of 15 U/ml of HumINS. The worms of the DAF-16 knock-down (B) and double treatment group (E) resulted in increased α-syn expression irrespective of the protective role of HumINS as compared to worms of the control group (A, D). Knocking down DAF-16 rendered ILS pathway inactive and the protective effect of HumINS in decreasing α-syn was not observed confirming the involvement of DAF-16 in HumINS action against α-syn expression. (**C**, **F**) Ggraphical representation for fluorescence intensity of α-syn::YFP as quantified using Image J software. ^***^ *p* < 0.001, Scale bar, 50 µm.

**Figure 5:**
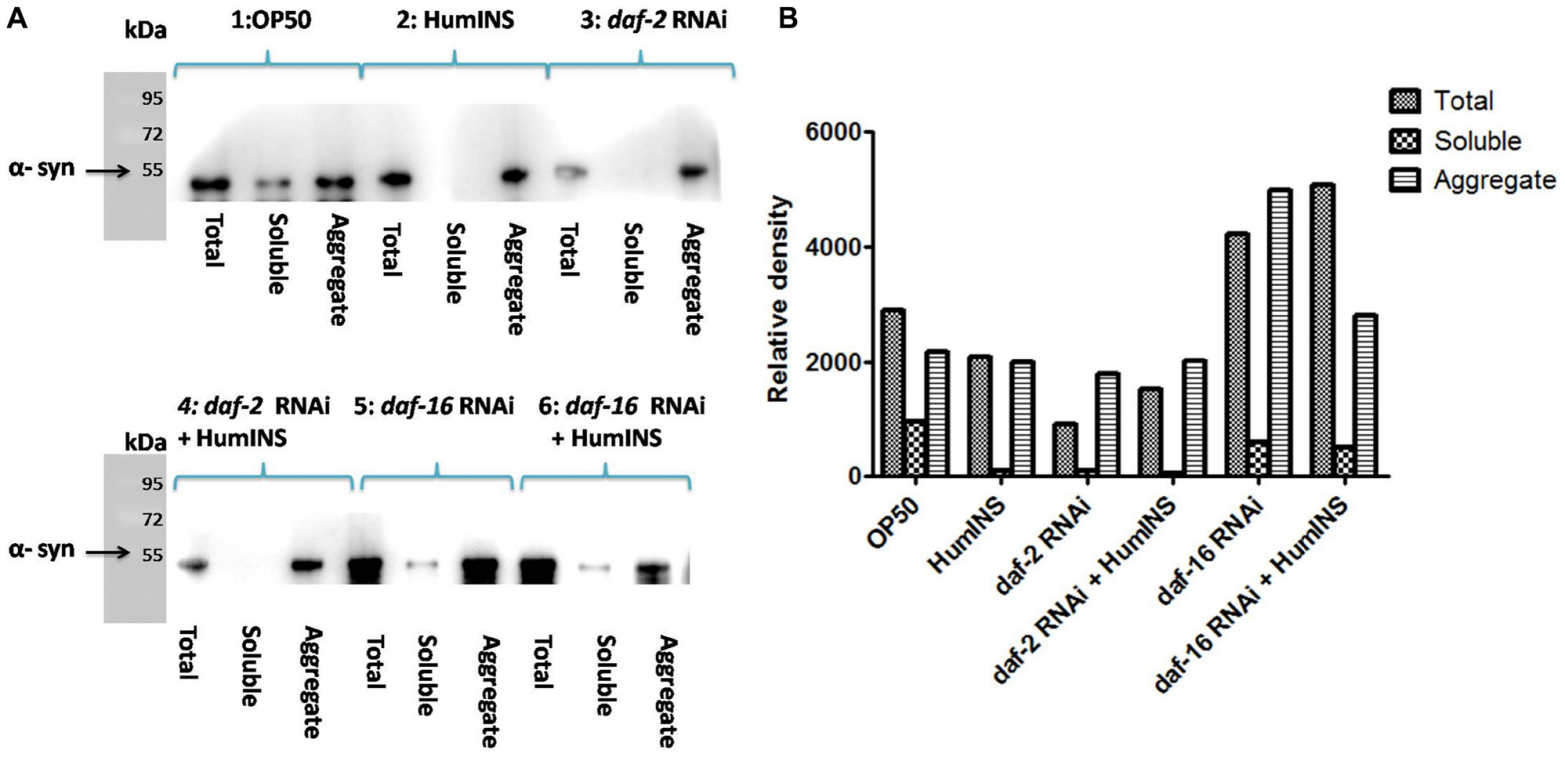
Western blot analysis of the level α-syn aggregation. A Western blot analysis of total protein, soluble and aggregateα-syn isolated from NL5901 transgenic worm expressing human α-syn::YFP (**A**). The worms were raised on OP50 diet (1), treatment of 15 U/ml HumINS (2), Under DAF-2 knock down (3), co-treatment of HumINS along with DAF-2 knockdown (4), Under DAF-16 knock down (5) and co-treatment of HumINS along with DAF-16 knockdown (6). (**B**) Represents the quantification of the band intensity by densitometry analysis using Image-J software.

### HumINS decreases aging-dependent aggregate accumulation

In further support of the α-syn aggregation at single time point, we wanted to study the age specific expression pattern of α-syn under the effect of insulin. Ageing, by far, adversely impacts the outcome of Parkinsonism, thus worsening of condition is seen as an affected individual ages. In order to examine this aspect, we performed time-kinetics experiment until late adult stage of the worms. Transgenic strain NL5901 that bears a Human α-syn was subjected to 15 U/ml human insulin treatments as described earlier. The worms from the control and treatment plates were examined for α-syn aggregates by fluorescent microscopy on day-5 and day-7 (an earlier time point of day-2 has already been described above). In this study, we observed that the ageing process itself, in the absence of HumINS i.e. in the control worms, leads to increase in number of α-syn aggregates. The α-syn expression was observed to be diffused on day-2 as evident from Figure 1. But the number and size of aggregates increased as the worm aged up to day-5 which is depicted in Figure 2A. In the later stages of ageing, the size of aggregates increased but number decreased (Figure 2B). Also, worms treated with 15 U/ml HumINS exhibited a significant decrease in aging-dependent α-syn aggregate accumulation at day-5 and day-7. The mean number of protein aggregates in control worms at day-5 was 113.8 ± 7.57 *N* = 13 arbitrary units and 67.00 ± 7.015 *N* = 13 in 15 U/ml HumINS treated worms (Figure 2E). While, the mean number of protein aggregates in control worms at day-7 was 72.00 ± 4.570 *N* = 16 arbitrary units and 35.06 ± 3.865 N = 18 in 15 U/ml HumINS treated worms (Figure 2E). A 1.69 and 2-fold reduction (*p* < 0.001) in α-syn protein aggregates was observed in 15 U/ml HumINS exposed worms at day-5 and day-7 respectively.

### α-syn aggregation is mediated via insulin like receptor, DAF-2 activity

In *C. elegans daf-2* encodes a receptor tyrosine kinase which is the ortholog of the HumINS/IGF receptor [30]. The fact that an apparent increase in ILS/IGF/AKT pathway activation in invertebrate model protects neurons against proteotoxicity, has been reported [31, 32]. Keeping this in mind we speculate that the HumINS is exerting its anti aggregation effect through deactivating ILS pathways which is further governed by DAF-2 insulin-receptor activation. This hypothesis was based on the finding of Pierce *et al.*, who in their research work reported that HumINS antagonizes DAF-2 ILS pathway [20]. Alternatively, HumINS could have also exerted its protective effect via other stress responsive pathways apart from ILS which may have resulted in the α-syn detoxification. To distinguish between these two possibilities, the very first question we asked was whether DAF-2 plays a role in α-syn aggregation in the *C. elegans* PD model via ILS pathway as the absence of DAF-2 would not result in alteration of α-syn::YFP expression. To answer this question, we carried out α-syn expression studies in NL5901 transgenic strain of *C. elegans*. A marked decrease in the α-syn::YFP expression under DAF-2 silenced condition was observed (Figure 3A, 3B). The mean fluorescence (GFP) intensity was 27.84 ± 1.03 *N* = 9 arbitrary units in control worms and 14.78 ± 0.74 *N* = 9 arbitrary units in DAF-2 knockdown worms (Figure 3C). A 1.88-fold decrease (*p* < 0.001) in α-syn protein expression was observed in *daf-2*RNAi worms. We also quantified α-syn aggregation level employing Western blotting. We observed that worms of *daf-2*RNAi treated group exhibited lower aggregation, lesser total α-syn as compared to the worms of control group (Figure 5).This outcome was expected as DAF-2 knockdown should have resulted in DAF-16 nuclear localization hence, transcription of stress responsive genes. Therefore, this data proves that the α-syn expression and aggregation is mediated via insulin-receptor, DAF-2 activity.

### HumINS acts as an antagonist for insulin-receptor DAF-2 in reducing α-syn aggregation and is also dependent on its activity

According to our proposed hypothesis, HumINS is exerting its anti aggregation affect through deactivating ILS pathway which is further governed by DAF-2 insulin-receptor activation. In order to answer this we further moved on to test out whether HumINS also involves DAF-2 receptor in their signalling process to decrease α-syn aggregation. For assaying this we carried out double treatment studies, we subjected the NL5901 transgenic strain of *C. elegans* to both DAF-2 knockdown along with co-treatment of HumINS and compared the α-syn expression and aggregation level with the control worms fed on OP50. Knocking down DAF-2 will render ILS pathway inactive and any protective effect of HumINS in decreasing α-syn aggregation will not be prominent/significant. In addition, if the intensity in the level of α-syn of double treatment will be identical as to that of level of α-syn as of DAF-2 knockdown only or in other words if there is no synergistic effect of HumINS treatment and DAF-2 knockdown then the only possible way for HumINS action is through DAF-2 only. This will negate the involvement of any other signalling based reduction in α-syn aggregation. In our experiments we observed a significant decrease in the α-syn expression in the worms co-treated with *daf-2* RNAi and HumINS (Figure 3D, 3E). The mean fluorescence (GFP) intensity was 27.84 ± 1.03 *N* = 9 arbitrary units in control worms and 16.60 ± 1.22 *N* = 9 arbitrary units in worms co-treated with *daf-2* RNAi and HumINS (Figure 3F). A 1.68-fold decrease (*p* < 0.001) in α-syn protein expression was observed in insulin-*daf-2* co-treated worms. The α-syn expression was similar to the worms in which only DAF-2 was knocked-down. Further, we went on to carry out quantification of α-syn aggregation level employing Western blotting, we observed that worms of *daf-2* silenced along with co-treatment of HumINS groups exhibited reduction in α-syn aggregate level. And the level of reduction was also similar for both *daf-2* silenced group and the group wherein *daf-2* was silenced along with co-treatment of HumINS (Figure 5). These similar effects may only be observed when DAF-2 was involved in the modulation of α-syn aggregation otherwise HumINS would have displayed its protective effect and a significant synergistic effect would have been observed. In addition to this, present experiment also advocates that HumINS also acts as the antagonist for DAF-2 receptor, as the only possible way for HumINS is deactivating DAF-2 receptor (as no other receptors are involved) that would lead to increase in DAF-16 expression and finally reduction in α-syn::YFP. Present assay indicates that HumINS involves DAF-2 in reducing α-syn and confirms the dependency of HumINS on DAF-2 activity.

### Stress-responsive FOXO transcription-factor DAF-16 is responsible for reduction in α-syn aggregation

The dephosphorylation of DAF-16 leads to expression of numerous chaperonic genes, increases stress resistance, and increases the life span of worms [33]. After the confirmation of the involvement of DAF-2 receptor in HumINS mediated reduction in α-syn aggregation in our previous results, we asked the question whether DAF-2 and HumINS mediated signalling involves DAF-16? This question was based on the assumption that there may be further branching in DAF-2 signalling in addition to the documented canonical DAF-2/DAF-16 signalling. In order to answer this we first confirmed whether DAF-16 has a role to play in α-syn aggregation in the *C. elegans* PD model via ILS pathway. If DAF-16 plays a role in α-syn::YFP expression then the absence of DAF-16 would result in alteration of α-syn::YFP expression. Concerning the mechanistic predicament we chose the loss of function studies of the DAF-16 in NL5901 transgenic strain of *C. elegans* by RNAi mediated gene silencing, we observed an increase in the α-syn expression pattern in *daf-16* silenced worms (Figure 4B) when compared to worms of control group (Figure 4A) which was expected because of the stress responsive role of DAF-16. The mean fluorescence (GFP) intensity was 21.28 ± 2.911 *N* = 8 arbitrary units in control worms and 35.72 ± 1.611 *N* = 8 arbitrary units in DAF-16 knockdown worms (Figure 4C). A 2.80-fold increase (*p* < 0.001) in α-syn protein expression was observed in *daf-16* RNAi worms. We also quantified α-syn aggregation level employing Western blotting. We observed that worms of *daf-16* RNAi treated group exhibited a higher aggregate and total α-syn from the worms of control group (Figure 5).

### The effect of HumINS on α-syn aggregation is dependent on the activity of FOXO transcription-factor DAF-16

To understand the mechanism behind the observed effect of reduction in α-syn aggregation, by HumINS, we studied *daf-16* gene that encodes *C. elegans* forkhead box O (FOXO) homologue and acts as a transcription-factor of the ILS pathway. We subjected the NL5901 transgenic strain of *C. elegans* to both DAF-16 knockdown along with co-treatment of HumINS and compared the α-syn expression and aggregation level with the control worms fed on OP50 as done in the case of combined treatment of DAF-2 and HumINS. We observed a significant increase in the α-syn expression in the worms co-treated with DAF-16 knockdown and HumINS (Figure 4E) when compared to control worms (Figure 4D). The mean fluorescence (GFP) intensity was 33.75 ± 0.9508 *N* = 11 arbitrary units in control worms and 43.55 ± 0.5651 *N* = 11 arbitrary units in worms co-treated with *daf-16 RNAi* and HumINS (Figure 4F). A 1.30-fold increase (*p* < 0.001) in α-syn protein expression was observed in *daf-16* co-treated worms. The α-syn aggregation in co-treated worms was also similar to the worms in which only DAF-16 was knocked-down as quantified using Western blotting (Figure 5). *daf-16* silenced worms and the worms in which *daf-16* was silenced along with co-treatment of HumINS, exhibited increase in α-syn aggregate level, and the level was similar for both *daf-16* silenced and *daf-16* silenced + HumINS treated worm populations, as can be seen in Figure 5, indicating that the HumINS did not influence the aggregation pattern of *daf-16* RNAi treatment. Therefore, the protective effect of HumINS against α-syn aggregation is mediated by DAF-16 transcription-factor in transgenic strain of *C. elegans* expressing human α-syn.

### *C. elegans* endogenous DAF-2 receptor antagonist INS-17 and INS-18 exhibited same effect on α-syn expression as that of HumINS

**Figure 6:**
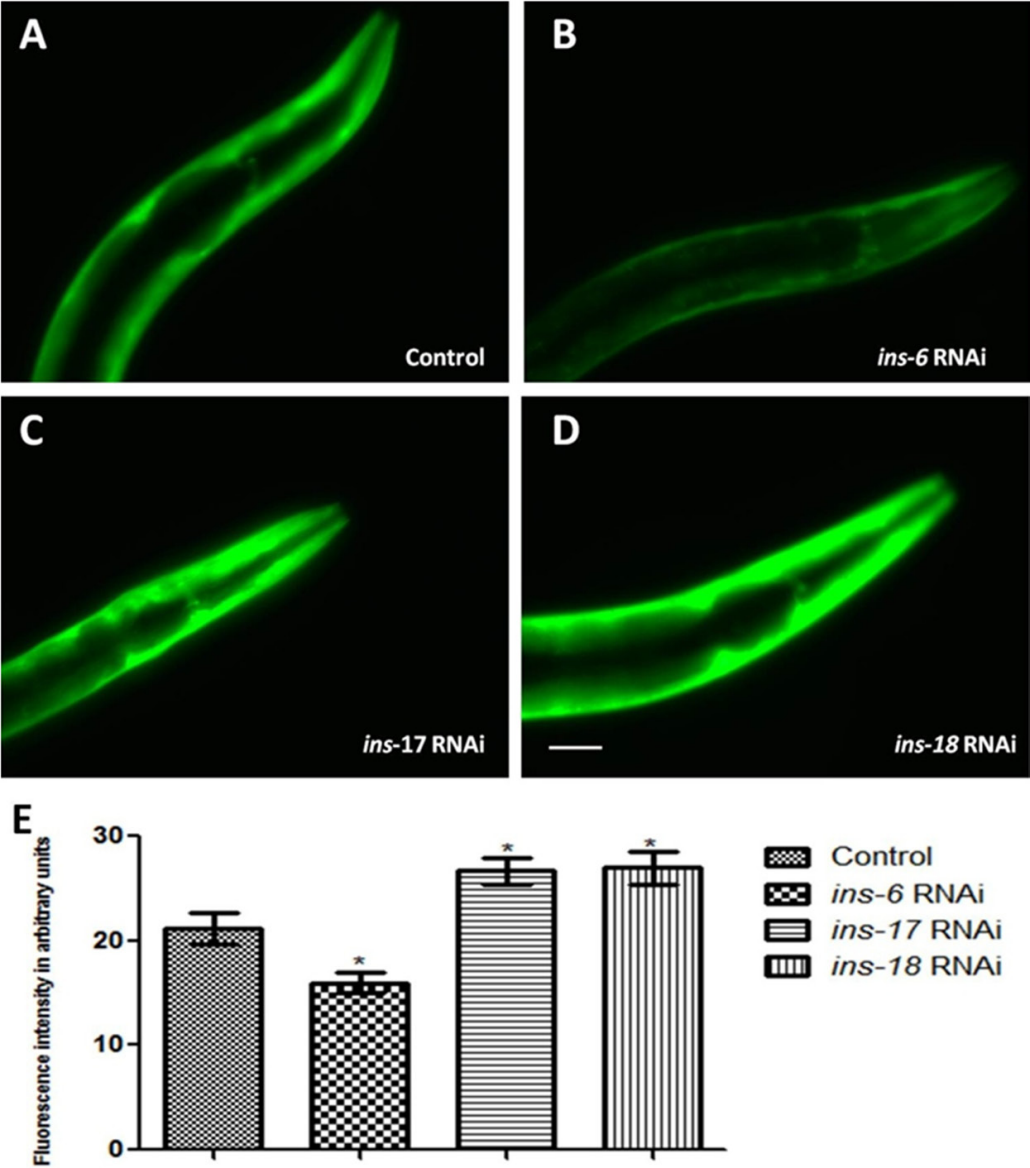
*C. elegans* DAF-2 receptor antagonist INS-17 and INS-18 and agonist INS-6 exhibited opposite effect on α-syn expression. (**A–D**) Fluorescent images represent knockdown studies of NL5901 transgenic strain of *C. elegans*, in which transgenic worm were: fed on OP-50 (control; A), bacteria expressing double stranded RNA against *ins-6* (B), bacteria expressing double stranded RNA against *ins-17* (C), and bacteria expressing double stranded RNA against *ins-18* (D)*. C. elegans* endogenous antagonist INS-17 and INS-18 also act as that of external insulin in modulating α-syn::YFP pattern and the effect is reversed when agonist INS-6 is used. (**E**) Graphical representation for fluorescence intensity of α-syn::YFP as quantified using Image J software. ^*^ *p* < 0.05, Scale bar, 50 µm. The image produced for publication was imaged on the ImageQuant LAS 4000 series used together with the ImageQuant LAS 4000 Control Software. No specific bands were handled individually or differently vs. that of any comparing bands. The bands for each image are from a single blot, handled uniformly including that for contrast/brightness. As mentioned, the ImageQuant LAS 4000 software was used to process the images in order to be better presentable for publication. This may have made background data brighter and created a corresponding shift in an image’s white point.

By partial characterization and reverse genetics approaches, insulin superfamily genes that bind to DAF-2 have been identified and classified within the cluster of *C. elegans ins* genes. Approximately, 37 putative insulin-like ligands have been reported that can regulate ILS pathway [20]. Those which can activate DAF-2 are characterized as “agonists” and which can deactivate DAF-2 as “antagonists”. Survival of the worms is favored under stress conditions by antagonism of *daf-2* signalling while activation of *daf-2* signalling benefits growth and reproduction. Depending upon the environmental conditions some INS proteins may be DAF-2 agonists, whereas others may be antagonists. *C. elegans* predicted insulin like peptide gene *ins-17* and *ins-18* which structurally resemble each other encode type-γ insulin like peptides, INS-17 and INS-18 respectively. INS-17 and INS-18 are reported to function as antagonists for DAF-2 receptor and are required for lifespan extension [34–36]. In order to answer whether endogenous *C. elegans* insulin like antagonist ligand can modulate the α-syn expression outcome in transgenic NL5901 strain like HumINS, we chose to study INS-17 and INS-18 loss of functions conditions. We carried out RNAi mediated gene silencing of *ins-17* and *ins-18* gene in transgenic strain expressing Human α-syn::YFP. We observed that DAF-2 receptor antagonist INS-17 or INS-18 knockdown resulted in increased α-syn expression (Figure 6C, 6D) as compared to control group (Figure 6A). The mean fluorescence (GFP) intensity was 21.19 ± 1.520 *N* = 7 arbitrary units in control worms, 26.65 ± 1.205 *N* = 8 arbitrary units in INS-17 knockdown worms and 26.96 ± 1.541 *N* = 7 arbitrary units in INS-18 knockdown worms (Figure 6E). A 1.26 and 1.27-fold increase (*p* < 0.05) in α-syn protein expression was observed in *ins-17* and *ins-18* RNAi worms, respectively. The reason behind this can be verily explained as RNAi of *ins-17* or *ins-18* resulted in reduced *ins-17* or *ins-18* mRNA level hence the binding of INS-17 or INS-18 with DAF-2 will be lesser thus encouraging basal DAF-16 phosphorylation leading to negative regulation of protective genes, in turn leading to increased α-syn expression. The observed data is in agreement with the antagonist effect of HumINS on α-syn aggregation performed earlier and concurs with the idea that DAF-2 antagonist may reduce α-syn abundance in transgenic *C. elegans* PD model.

### *C. elegans* endogenous DAF-2 receptor agonist INS-6 exhibited opposite effect on α-syn expression than that of HumINS

*C. elegans* predicted insulin like peptide gene *ins-6* encodes a type-beta insulin-like molecule and is expressed throughout its lifespan, and in some neurons at early developmental stages [20]. INS-6 structurally resembles HumINS and is reported to bind and activate the HumINS receptor thus INS-6 acts as an agonist for ILS pathway in *C. elegans* [34, 37]. To determine whether *C. elegans* endogenous DAF-2 receptor agonist shows the opposite effect on α-syn expression pattern as that of *C. elegans* DAF-2 receptor antagonist in transgenic NL5901 strain we carried out INS-6 loss of function studies. We observed that DAF-2 receptor agonist INS-6 knockdown (Figure 6B) resulted in reduced α-syn expression when compared to control group (Figure 6A). The mean fluorescence (GFP) intensity was 21.19 ± 1.520 *N* = 7 arbitrary units in control worms, 15.95 ± 0.9726 *N* = 7 arbitrary units in INS-6 knockdown worms (Figure 6E). A 1.33-fold decrease (*p* < 0.05) in α-syn protein expression was observed in *ins-6* RNAi worms. This outcome was expected as the DAF-2 agonist would ideally act opposite as that of DAF-2 antagonists. RNAi of INS-6 resulted in reduced *ins-6* mRNA level hence there will be a reduced binding of INS-6 and DAF-2 while DAF-16 will undergo nuclear localization leading to expression of protective/stress responsive genes and decrease in α-syn abundance. The observations are consistent with previous findings [20] and strengthen the data obtained in the previous section, suggesting that antagonists negatively affect and agonists promote α-syn aggregation via DAF-2/DAF-16 signalling pathway in transgenic PD model of *C. elegans.*

### Computational analysis predicts different binding sites for agonists and antagonists on DAF-2 receptor

**Figure 7:**
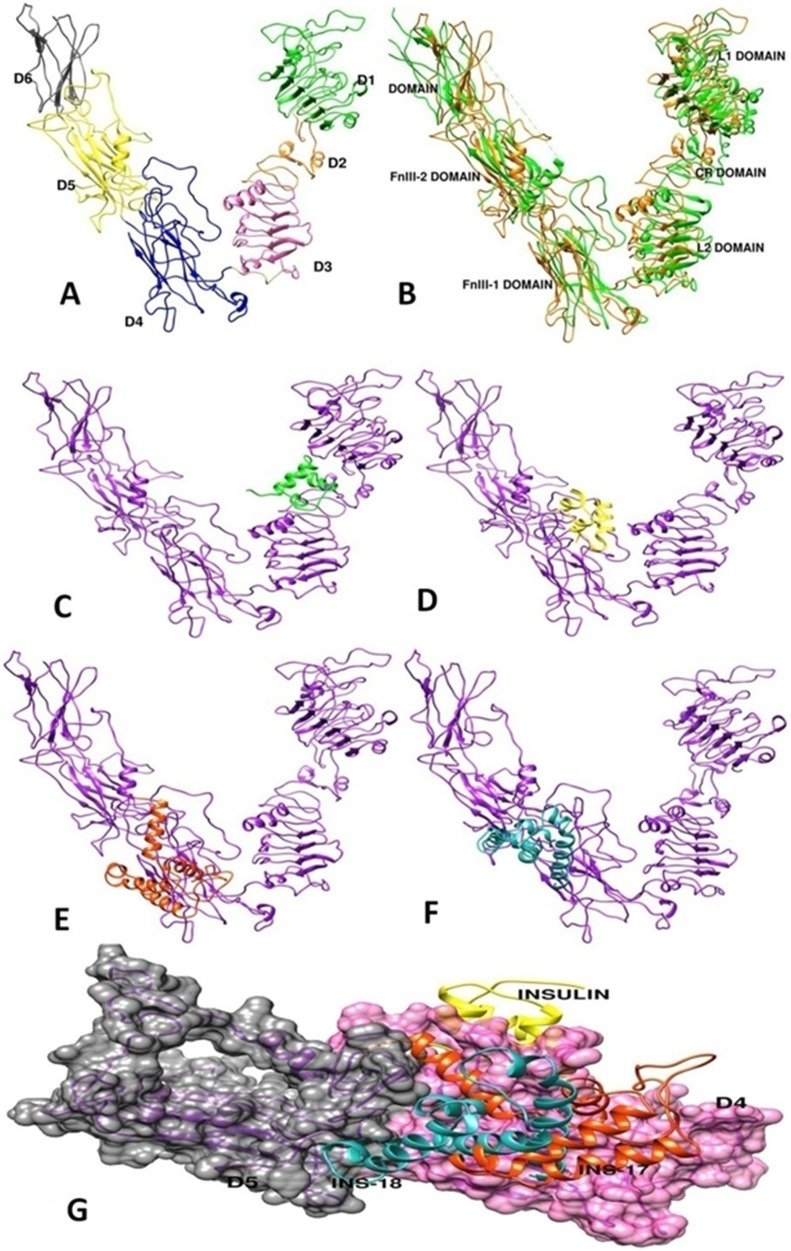
Three-dimensional structure construction of *C. elegans* DAF-2 insulin-receptor and molecular docking studies of agonist and anatagonist on modelled DAF-2 structure. (**A**) Indicatingmodelled structure of DAF-2 receptor using Modeller version 9.14 having six domains namely D1-D6. (**B**) Indicating overlapped structure of DAF-2 protein (green) with its homolog i.e. human insulin-receptor ectodomain having PDBid 3LOH (orange). Domain nomenclature was as per PDBid-3LOH. (**C**) Illustrates the potential binding site of INS-6 on ectodomain of DAF-2 protein, occuring between D1, D2 and D3 domain. (**D**, **E**, **F**) Indicate potential common binding site of HumINS, INS-17, INS-18 peptides on ectodomain of DAF-2 protein. (**G**) Represents the merged image of different binding site of antagonist insulin, INS-17 and INS-18 on DAF-2 protein occuring between D4 and D5 domain respectively.

*C. elegans* DAF-2 receptor protein structure is not solved till now, thus in order to predict the binding sites of DAF-2 agonists and antagonists, DAF-2 insulin binding domain was modeled using Modeller version 9.14. After establishing the mechanism of action of HumINS via ILS in worms we speculated that the difference in activity of the agonists and antagonists towards DAF-2 activation may be due to their different structure, if any. Dissimilarity, in structure may further be responsible for different binding sites hence resulting in different biological activity in the same receptor. For answering this, homology models were generated using structure of the human insulin-receptor ectodomain (PDB-3LOH and 2DTG). PDB - 3LOH and 2DTG showed 31% identity with query sequence. Best DAF-2 structure model indicated 97.8% residues in favored region, addition allowed region and generously allowed region while 2.1% residues in disallowed region. The modeled DAF-2 protein structure contained 6 domains comparable to L1, CR, L2, FnIII-1, FnIII-2, FnIII-3 domains of human insulin-receptor ectodomain [38] and were labeled as domains D1, D2, D3, D4, D5 and D6 in Figure 7A, 7B. Domain D1 and D3 was found to be leucine rich while D2 was cystiene rich as compared to its homolog, i.e., ectodomain of human insulin-receptor. Modeled DAF-2 protein showed low RMSD of 1.2 Å with its homolog. The modeled DAF-2 receptor antagonist namely INS-17 and INS-18 structures were found to have 100% residues in favored region, addition allowed region, generously allowed region and none in disallowed region (Figure 7E, 7F). Subsequently, the modeled DAF-2 receptor protein was docked with DAF-2 agonist (INS-6) (Figure 7C) and antagonists (HumINS (Figure 7D), INS-17 (Figure 7E), INS-18 (Figure 7F)). The computational docking studies using ZDOCK indicated that largest cluster of docked poses of INS-6 [PDB-2KJI] protein was formed in site-1 binding pocket of DAF-2, which is constituted by the D1, D2 and D3 domains (Figure 7G). On the contrary, the docking studies with DAF-2 antagonist (HumINS [PDB-4F4V], INS-17, INS-18) indicated that the largest binding cluster of all antagonists were present in binding site-2 which is formed by the participation of D4 and D5 domains interface of DAF-2 protein. ZDOCK score was 22.54 for INS-6 and 21.92, 25.4, 25.86 for insulin, INS-17 and INS-18 respectively. Some of the previous findings [37, 39, 40] also indicated that agonists may bind to site 1 of DAF-2. Previously reported work on human insulin-receptor [10–12] also proposes that Fibronectic domain FnIII-1, FnIII-2 of first monomer (corresponding to D4 and D5 domains of DAF-2 respectively) interacts with L1, CR, L2 domain of second monomer (corresponding to D1, D2, D3 domains of DAF-2 respectively) which upon dimerization forms a stable insulin binding site-1 and FnIII-1(corresponding to domain-4), FnIII-2 (corresponding to domain-5) forms site-2 in human insulin-receptor [39–41]. Our findings suggest that domain D1, D2 and D3 (site-1) may form agonist (INS-6) binding pocket while D4 and D5 domains (site-2) are predicted to form an antagonist (HumINS, INS-17 and INS-18) binding pockets.

## DISCUSSION

**Figure 8:**
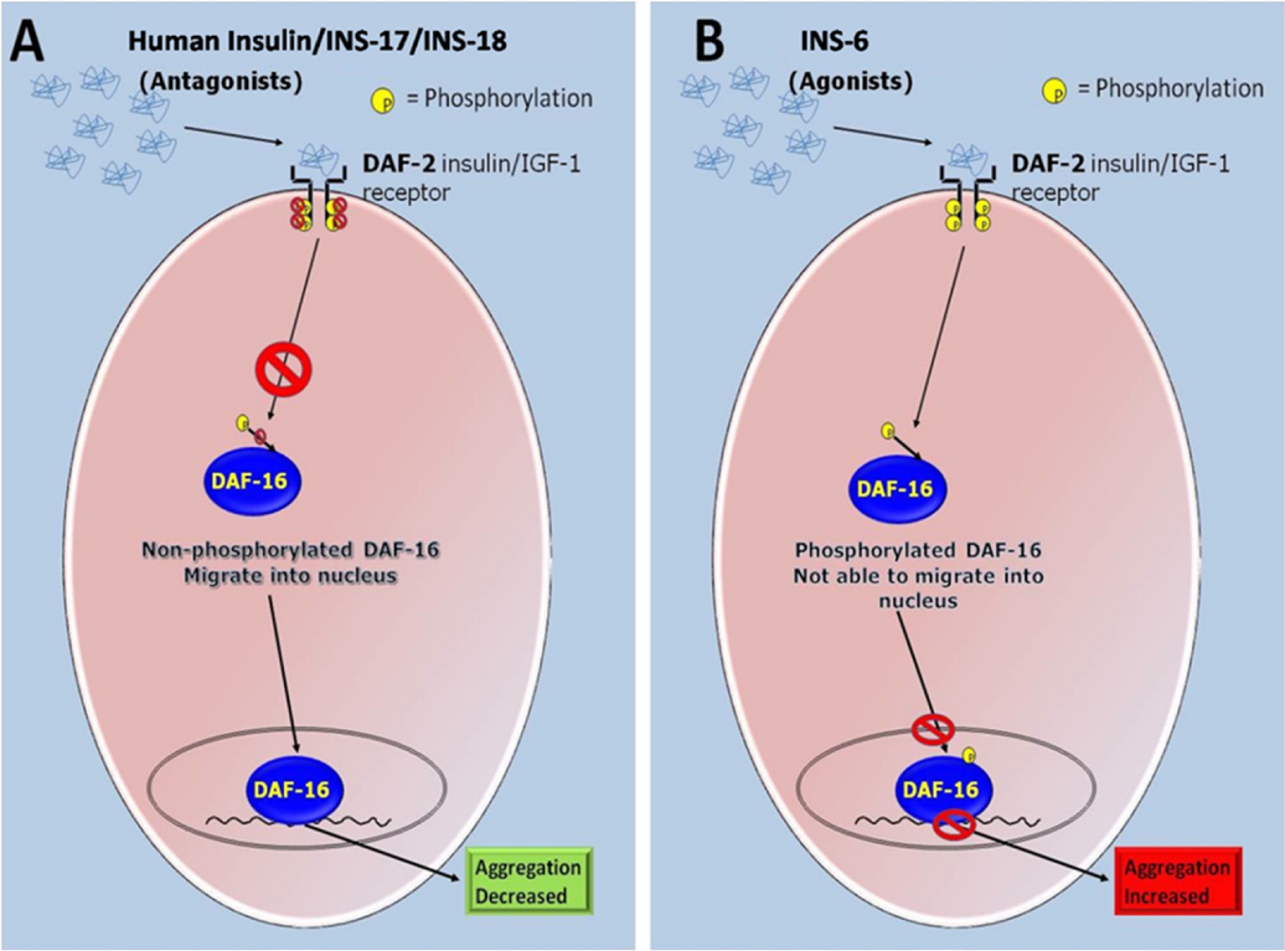
Schematic representation of the proposed roles of the agonist and antagonist of DAF-2 mediated ILS pathway of the *C. elegans* in modulating α-syn aggregation. (**A**) DAF-2 deactivation by antagonists inhibits phosphorylation of DAF-16 hence promoting its nuclear entry and transcription of the stress responsive genes resulting in decreased α-syn aggregation. (**B**) DAF-2 receptor activates a cascade of protein kinases which ultimately terminates at the phosphorylation of downstream transcription-factor DAF-16 by AKT protein kinase. DAF-2 activation by agonist phosphorylate DAF-16 to prevent its nuclear entry and transcription of the stress responsive genes are switched off resulting in increased α-syn aggregation.

Insulin-signalling is the primary pathway that regulates glucose and fat metabolism. In its canonical pathway insulin activates its receptor molecule which happens to be a tyrosine kinase, through addition of a phosphate moiety. Activation of insulin-receptor starts a downstream cascade of various adaptors that incorporate Akt/PKB and the PKCζ cascades which further induce processes like glycogen & fatty acid synthesis, cell survival, growth and inhibit apoptotic transcriptional factors, gluconeogenesis [42, 43]. A potential linkage of insulin signalling alterations to cognitive function has been suggested. By employing *C. elegans* transgenic strain NL5901 which expresses human α-syn in the body wall muscle we first, analyzed the α-syn expression and aggregation after treatment of HumINS. We chose HumINS as it is known to bind with receptors of *C. elegans* orthologue INS proteins and vice versa, thereby leading downstream signaling [20, 37]. We observed that HumINS treatment decreases α-syn aggregation. A potential linkage of HumINS with α-syn aggregation prompted us to study the effect of DAF-16/DAF-2 knockdown on α-syn aggregation by employing reverse genetics approaches. In our studies, worms in which DAF-16 was shut down, exhibited an increase in α-syn aggregation and worms in which DAF-2 was shut down, exhibited a decrease in α-syn aggregation. The data obtained is consistent with previous findings and also establishes the governance of α-syn aggregation via HumINS involves DAF-16 molecule, we carried out double treatment studies in which both DAF-16 was knocked-down along with HumINS treatment. We observed a significant increase in the α-syn aggregation in the worms co-treated with DAF-16 RNAi and HumINS. The α-syn aggregation was similar to the worms in which only DAF-16 was knocked-down. This similar effect may only be observed when DAF-16 was involved in the modulation of α-syn aggregation otherwise HumINS should display its protective effect. Similarly we also carried out double treatment studies in which DAF-2 was knocked-down independently and along with HumINS treatment. We also observed a significant decrease in the α -syn aggregation in the worms co-treated with DAF-16 RNAi and HumINS and the effect was not synergistic with HumINS treatment but aggregation was similar to the worms in which only DAF-16 was knocked-down. This establishes the involvement of DAF-2 in the α-syn aggregation. Next we moved on to decipher the effect of *C. elegans* endogenous insulin like ligands on α-syn expression. We employed INS-6 as agonist and INS-17 and INS-18 as antagonists for α-syn studies. Our results suggest that endogenous antagonist insulin like molecule of *C. elegans* also acts as that of external insulin and the effect is reversed when agonists are used which is pictorially depicted in Figure 8. This clearly indicates that insulin-receptor antagonist in *C. elegans* plays a protective role in α-syn mediated toxicity and targeting IS in mammals may prove to be beneficial in the treatment of life threatening NDs. Further, we modeled the 3D structure of the DAF-2 receptor protein by the use of computational tools. The modeled structure of DAF-2 protein was predicted to have 6 domains that were similar to the previously reported X-ray structure of the ectodomain of Human insulin-receptor protein. Also due to non-availability of structure of DAF-17 and DAF-18, their 3D structures were also modeled using the same approach. Lastly, the agonist (INS-6) and antagonists (HumINS, INS-17 and INS-18) were docked on the modeled DAF-2 structure. The computational findings indicate that agonist binds at a different site than the antagonists on DAF-2 protein. The different binding pocket for agonists and antagonist may be responsible for their differential activity towards activation or deactivation of the ILS pathway (DAF-2 pathway) in nematode *C. elegans*. The computational studies revealed a possible mechanism of action of agonists and antagonist with respect to α-syn aggregation via DAF-2 receptor.

## MATERIALS AND METHODS

### *C. elegans* culture and maintenance

Various strains of *C. elegans* used in this study were cultured using standard techniques [44]. Standard bacterial food *Escherichia coli* (*E. coli*) strain OP50 was fed to *C. elegans* except for the gene knockdown (RNAi) experiments wherein the genetically engineered HT115 *E. coli* strains have been employed. Strains were propagated at 22°C on nematode growth medium (NGM) solidified on 90 mm petri plates and seeded with 500 µl of *E. coli* as food source.

### Age synchronization

For age synchronized populations of the nematodes, reproductively mature *C. elegans* were washed from 3–4 petri plates using 10 ml M9 buffer and transferred to 15 ml centrifuge tube and subjected to centrifugation at 1300 rpm for 2 min. To clear off any adhering bacteria, the worm pellet was again suspended in 10 ml M9 and washed thrice. To the worm pellet, 5 ml of 1 M sodium hydroxide solution and 2 mL of sodium hypochlorite (together known as axenizing solution) was added, followed by gentle vortexing until worm bodies were dissolved and eggs were released into the solution. The released eggs were allowed to settle down by spinning at 1300 rpm for 5 min. and washed twice with M9 buffer. The isolated eggs were placed onto the bacterial lawn of experimental plates.

### *C. elegans* strains

The wild-type strain N2 (Bristol), transgenic-strain NL5901 were employed in this study. NL5901 (pkIs2386 [unc-54p::α-syn::YFP + unc-119(+)]) expresses Human α-syn protein fused to Yellow Fluorescent Protein (YFP). Human α-syn is expressed in the body wall muscle cells of the nematode driven by unc-54 promoter [45]. All the strains were obtained from *Caenorhabditis* Genetics Center (University of Minnesota, Minneapolis, MN).

### Preparation of HumINS treatment plates

Biphasic isophaneHumINS under the trade-name Huminsulin^®^ was mixed with OP50 or HT115 *E. coli* bacterial strain expressing double-stranded RNA (homologous to the target gene) to obtain a concentration of 10 U/ml and 15 U/ml. 500 µl of the HumINS-bacterial suspension was seeded onto NGM or NGM-IPTG plates, dried and incubated overnight at 37°C. The *C. elegans* embryos were placed onto these plates and incubated at 22°C for 48 hrs.

### RNAi induced gene silencing

For achieving RNAi mediated gene silencing standard feeding protocol was followed as described previously [35]. The bacterial clone, expressing dsRNA targeted for *C. elegans daf-16, ins-6, ins-17, ins-18* was obtained from AhringerRNAi library purchased from SA Biosciences (Cambridge, UK). And bacterial clone, expressing dsRNA targeted for *C. elegans daf-2* was constructed. Briefly, *E. coli* strain harbouring the appropriate dsRNA expressing vectors were grown in luria broth containing 50 μg/ml ampicillin and incubated in shaking incubator at 37°C overnight. Then 500 µl of bacterial culture were seeded onto NGM plates containing 5 mM isopropyl β-d-thiogalactoside (IPTG) and 25 mg/L carbenicillin followed by an overnight incubation at 37°C to induce expression. *C. elegans* embryos were transferred onto these plates and incubated at 22°C for 48 hrs for further studies.

### Plasmid constructs

Plasmids were constructed using standard cloning techniques as described previously [46]. A short segment of *daf-2* gene (sequence number Y55D5A.5) was amplified using standard PCR with primer set bearing sacI and kpnI endorestriction sites. The PCR condition was: 50 ng of *C. elegans* genomic DNA, 400 nanomolar forward and reverse primer, 10 mM dNTPs followed by incubation at 95°C for 10 min (1 cycle), 94°C for 30 sec, 55°C for 30 sec., 72°C for 30 sec (30 cycle), and 72°C for 5 min (1 cycle). The amplified *daf-2* gene fragment was further sub cloned in TA vector (pCR^®^ 2.1 vector) (invitrogen cat no. 450046) and then cloned into Timmons and Fire feeding vector L4440 under standard conditions and was finally transformed into HT115 (DE3), an RNase III-deficient *E.coli* strain with IPTG inducible T7 polymerase activity.

*daf-2* Forward Primer: GAG CTC TGT CTC AGT AAC GGC GAC CTC T (Tm 59.2°C).

*daf-2* Reverse Primer: GGT ACC CGT TGG CAC ATC ATT CTC TCG (Tm 57.7°C).

### Western blotting of α-syn

Nematode populations from treatment plates were harvested using M9 buffer, and further washed thrice with M9 buffer and twice with PBS. For preparation of total protein extract, worms were suspended in 200 μl phosphate buffer saline (PBS) containing protease inhibitor cocktail (Thermo Scientific™ Halt™ Protease Inhibitor Cocktail). For the homogenization of the worms, sonication was performed for 3 min (15 sec pulse on and 15 sec pulse off) in sonicator (Misonix S4000) using microtip. The protein lysate was centrifuged at 16,000 × g for 30 min at 4°C to clear the worm debris and supernatant was used further. Using Bradford analysis protein concentration was determined for native lysates which were further used to fractionate soluble forms from the aggregated forms by centrifugation of 100 μl protein samples at 500 g for 15 min at 4°C using a membrane based filter (MRCF0R100, Millipore). This centrifugal filter enables the concentration of biomolecules with a molecular weight cut-off of 100 kDa. After centrifugation, the volume of filtrate (soluble) and unfiltered (aggregated) fractions was made up to 100 µl using PBS. 15 µg of total protein fraction, soluble fraction and aggregated fraction were loaded based on the total protein concentration (calculated previously). This ensured that the amount loaded for soluble and aggregate resemble their concentration in native extract. After that 12% sodium dodecyl sulfate Polyacrylamide Gel Electrophoresis (SDS-PAGE) was performed. Western blot analysis was performed using standard procedures as described [47]. For detection, the Antibody against α-syn, Anti-Aggregate α-syn Antibody, clone 5G4 (MABN389, Millipore) at 1:1000 dilutions was used. Anti-Mouse (Abcam, ab97046) at 1:5000 was used as the secondary antibody. Bands were detected using Super Signal West Pico Chemiluminescent Substrate (cat # 34080, Thermo Scientific). The densitometric analysis of the western blot bands was performed by gel analysis of Image J software (Image J, National Institute of health, Bethesda, MD, USA).

### Image acquisition

The images of the live transgenic worms expressing YFP or GFP were acquired following standard methodology. Worms were washed thrice with M9 buffer and immobilised using 100 mM (final concentration) sodium azide (Sigma, cat No. 71289) and mounted on an agar padded glass slide. Fluorescent microscope Carl Zeiss Axio Imager M2 was used with 20x or 40x objective equipped with AxioCam digital camera. A constant exposure time was followed for excitation while acquiring each image across control and various treatment groups. The expression level of α-syn::YFP was quantified using image J software (Image J, National Institutes of Health, Bethesda, MD, USA) by measuring florescence intensity. Quantification of α-syn::YFP was done by assaying an identical anatomical region across all subjects of the study. The fluorescence intensity of area between the mouth and posterior pharynx was measured and plotted in arbitrary units for each group. The microscope is controlled by ZEN2010 image processing software. For error free quantification across different planes of the Z-axis of nematodes, Z-stacking was carried out in the FITC channel.

### Age-dependent aggregate accumulation assay

Nematodes were raised on treatment plates until day 2 of adulthood. Roughly 300 worms were transferred each day on freshly prepared treatment plates in order to avoid mixing of generations. The worms were harvested from the treatment plates on day 5 and day 8 of adulthood. The harvested worms were washed and images of the transgenic strain were acquired in Z-stacking mode. For the quantification of the aggregates, method described previously was followed [48]. The number of α-syn aggregates was counted in the head region spanning from mouth to anterior pharynx using DotCount software (Copyright (c) 2006–2015 Martin Reuter). Aggregates were defined as bright GFP puncta like structures with boundaries distinguishable from surrounding fluorescence on all sides (number of dots in an image). Same size threshold was selected for analysis of each image.

### Statistical analysis

All experiments were performed in triplicate and within each group 7–10 images were analyzed for YFP or GFP expression level. The data sets are reported as mean ± standard error of the mean. The level of significance between different groups was compared statistically by employing Student’s *t* test using GraphPad prism 5 software packages.

### Computational studies

*C. elegans* insulin like receptor DAF-2 isoform-b (accession number NP_001122734.2) was chosen for the computational analysis and for 3D-structural investigation. Firstly, suitable templates were searched using BlastP tool [49] against protein data bank [50]. Homologs having good structural identity were retrieved; after that homology modeling approach was utilized in order to build 3D-protein models using structural information of the retrieved homologs as described previously [51]. Models were generated using Modeller 9.14 [52] and best 20 models with top DOPE score were validated on the basis of Ramachandran Plot in predicting structure using PROCHEK server [53]. UCSF Chimera [54] was used for the model visualization and other structural analysis. Due to lack of homologs having structure similarity to INS-17 and INS-18, *ab-initio* approaches [55, 56] were used to build their 3D-protein models using Robetta server [57]. Amongst the various models predicted by the server, best model was selected on the basis of Ramachandran Plot using PROCHEK server. Docking studies of INS-6, INS-17, INS-18, HumINS and DAF-2 protein were carried out using ZDOCK module of DISCOVERY STUDIO-4.

## CONCLUSIONS

HumINS treatment lowered the α-syn aggregation via DAF-2/DAF-16 pathway by acting as an antagonist for DAF-2 receptor. DAF-2 agonist (INS-6) and antagonists (INS-17 and INS-18) also resulted in a similar effect on α-syn aggregation. Computational studies revealed different binding pocket for agonists and antagonist and it may be responsible for the differential activity towards ILS activation. This opens avenues for the structure related targeting of ILS pathway, which may lead to interesting outcome towards treating PD in the near future.
